# Biofilm Forming Antibiotic Resistant Gram-Positive Pathogens Isolated From Surfaces on the International Space Station

**DOI:** 10.3389/fmicb.2019.00543

**Published:** 2019-03-19

**Authors:** Lydia-Yasmin Sobisch, Katja Marie Rogowski, Jonathan Fuchs, Wilhelm Schmieder, Ankita Vaishampayan, Patricia Oles, Natalia Novikova, Elisabeth Grohmann

**Affiliations:** ^1^Life Sciences and Technology, Microbiology, Beuth University of Applied Sciences, Berlin, Germany; ^2^Institute of Biology, University Freiburg, Freiburg, Germany; ^3^Institute of Biomedical Problems (IBMP), RAS, Moscow, Russia

**Keywords:** antimicrobial surface, gram-positive human-pathogenic bacteria, antibiotic resistance, biofilm, conjugative transfer, International Space Station, hostile environment

## Abstract

The International Space Station (ISS) is a closed habitat in a uniquely extreme and hostile environment. Due to these special conditions, the human microflora can undergo unusual changes and may represent health risks for the crew. To address this problem, we investigated the antimicrobial activity of AGXX®, a novel surface coating consisting of micro-galvanic elements of silver and ruthenium along with examining the activity of a conventional silver coating. The antimicrobial materials were exposed on the ISS for 6, 12, and 19 months each at a place frequently visited by the crew. Bacteria that survived on the antimicrobial coatings [AGXX® and silver (Ag)] or the uncoated stainless steel carrier (V2A, control material) were recovered, phylogenetically affiliated and characterized in terms of antibiotic resistance (phenotype and genotype), plasmid content, biofilm formation capacity and antibiotic resistance transferability. On all three materials, surviving bacteria were dominated by Gram-positive bacteria and among those by *Staphylococcus, Bacillus* and *Enterococcus* spp. The novel antimicrobial surface coating proved to be highly effective. The conventional Ag coating showed only little antimicrobial activity. Microbial diversity increased with increasing exposure time on all three materials. The number of recovered bacteria decreased significantly from V2A to V2A-Ag to AGXX®. After 6 months exposure on the ISS no bacteria were recovered from AGXX®, after 12 months nine and after 19 months three isolates were obtained. Most Gram-positive pathogenic isolates were multidrug resistant (resistant to more than three antibiotics). Sulfamethoxazole, erythromycin and ampicillin resistance were most prevalent. An *Enterococcus faecalis* strain recovered from V2A steel after 12 months exposure exhibited the highest number of resistances (*n* = 9). The most prevalent resistance genes were *ermC* (erythromycin resistance) and *tetK* (tetracycline resistance). Average transfer frequency of erythromycin, tetracycline and gentamicin resistance from selected ISS isolates was 10^−5^ transconjugants/recipient. Most importantly, no serious human pathogens such as methicillin resistant *Staphylococcus aureus* (MRSA) or vancomycin-resistant Enterococci (VRE) were found on any surface. Thus, the infection risk for the crew is low, especially when antimicrobial surfaces such as AGXX® are applied to surfaces prone to microbial contamination.

## Introduction

The International Space Station is an isolated habitat in a hostile environment. Microgravity, solar and cosmic radiation alter the immune-regulatory responses of the crew rendering them more susceptible to bacterial infections (Sonnenfeld, 2005; Crucian et al., 2008; Guéguinou et al., 2009). The microorganisms in the spaceship are human-derived; they originate from the crew and helpers who prepare the mission. The spaceship provides a special environmental niche for microorganisms, which directly or indirectly affect the health, safety or performance of the crew (Taylor, 2015). Microgravity can affect the virulence (Nickerson et al., 2004; Wilson et al., 2007; Rosenzweig et al., 2010; Crabbé et al., 2011), growth kinetics (Klaus et al., 1997; Kacena et al., 1999; Nickerson et al., 2004) and biofilm formation of microorganisms (Mauclaire and Egli, 2010). To assess the risk microorganisms pose to astronauts, the composition and properties of microbial communities in spaceships were analyzed. Two hundred and thirty-four bacterial and fungal species were found on the MIR space station, among those strong biofilm formers. *Staphylococcus* spp., followed by *Bacillus* spp. and *Corynebacterium* spp. were abundant in air as well as in surface samples (Novikova, 2004; Novikova et al., 2006). Schiwon et al. (2013) analyzed ISS samples from air and crewmembers in-flight and post-flight. *Bacillus* spp., *Staphylococcus* spp. and *Enterococcus* spp. were the most prevalent. 75.8% of the isolates exhibited resistance to one or more antibiotics. Corresponding resistance genes were found in 86% of the antibiotic-resistant bacteria. In 86.2% of the isolates horizontal transfer genes were detected. Eighty-three percent of the isolates were able to form biofilms (Schiwon et al., 2013).

Under spaceflight conditions, bacteria were shown to exhibit enhanced secondary metabolite and extracellular polysaccharide production as well as enhanced biofilm formation (Mauclaire and Egli, 2010; Vukanti et al., 2012). In space, the cell wall of *S. aureus* was significantly thicker than in the same strain grown on Earth (Novikova et al., 2006; Taylor, 2015). Various bacteria exhibited enhanced virulence, increased antibiotic resistance and differential gene expression under space conditions (Horneck et al., 2010; Yamaguchi et al., 2014; Taylor, 2015). Thus, these bacteria could spread their virulence and/or antibiotic resistance genes through horizontal gene transfer (HGT) and turn harmless bacteria into potential pathogens.

HGT is mediated by mobile genetic elements (MGEs), such as conjugative plasmids, conjugative transposons, integron-specific gene cassettes, or phages that are able to facilitate their own transfer. Plasmid-mediated HGT plays a primordial role in the emergence of new pathogens (Frost et al., 2005; Garbisu et al., 2018). Schiwon et al. (2013) found conjugative plasmids in bacterial isolates from the ISS and could demonstrate that some of these strains were able to transfer their antibiotic resistance genes to other bacteria. The HGT rate was shown to be higher in microbial biofilms than in planktonic cultures (Holmes et al., 2015). Biofilms represent a protected mode of microbial growth and confer significant survival advantages in hostile environments (Li et al., 2007; Thallinger et al., 2013). Thus, biofilm forming organisms show increased resistance to antibiotics, either due to decreased penetration of the antibiotic through the biofilm matrix or due to expression of more complex biofilm-specific resistance mechanisms.

Multiple antibiotic resistant and strong biofilm forming *Staphylococcus* and *Enterococcus* isolates detected on the ISS could pose an increased health risk on the crew (Schiwon et al., 2013). Several studies report, that bacteria from astronauts in-flight were more resistant to antibiotics due to enhanced biofilm formation or changes in cell morphology, e.g., thicker cell walls than isolates obtained from the same individuals either pre- or post-flight. As medical aid on the ISS is restricted, there is an urgent need for new antimicrobial materials, which can be used there to prevent infections by multi-resistant biofilm forming bacteria.

Heavy metals, e.g., copper and silver, have been known for a long time to possess antimicrobial activity. Silver was officially approved as an antimicrobial agent in the twentieth century (Chopra, 2007; Schäberle and Hack, 2014; Guridi et al., 2015; Vaishampayan et al., 2018). However, after the discovery of antibiotics the use of metals to combat bacterial infections has declined (Chopra, 2007; Grass et al., 2011). Later on, due to the increased occurrence of antibiotic resistant pathogens, silver and copper have again found widespread use, both in medicine and in everyday life (Maillard and Hartemann, 2012; Warnes and Keevil, 2013; Schäberle and Hack, 2014). These metals are easy to use as coatings on a variety of substrates and have a lethal effect on bacteria and fungi via the so-called contact killing (Grass et al., 2011). Silver is one of the best-studied bactericidal agents in water supplies (Russell and Hugo, 1994; Rohr et al., 1999; Vonberg et al., 2008; Vaishampayan et al., 2018). However, as occurred with antibiotics, bacteria have also developed resistance mechanisms against silver (Gupta et al., 1999). Like the excessive use of antibiotics, the extended use of silver is questioned due to its toxicity to the environment as well as to the human body (Landsdown, 2010). Plain ruthenium is not applied as antibacterial agent, but antibacterial activity has been demonstrated for ruthenium(II) polypyridyl complexes (Bolhuis et al., 2011; Li et al., 2011, 2015).

Due to the increasing resistance of bacteria to both antibiotics and commonly used antimicrobial metals, there is an urgent need to develop new approaches to combat bacterial infections. A new antimicrobial surface coating is AGXX® consisting of micro-galvanic elements of the two noble metals, silver and ruthenium, surface-conditioned with ascorbic acid (Vaishampayan et al., 2018). Both metals can be galvanically applied to diverse surfaces such as stainless steel, plastics, or cellulose fibers. The coating proved to be active against both Gram-positive and Gram-negative bacteria, but also against filamentous fungi, yeasts and some viruses (Guridi et al., 2015; Landau et al., 2017a,b; Vaishampayan et al., 2018). Recently, we demonstrated that it efficiently inhibits the growth of MRSA (Vaishampayan et al., 2018). The postulated mode of action is based on the formation of reactive oxygen species, particularly superoxide anions (Meyer, C., personal communication), which affect biomolecules, such as nucleic acids, proteins, and lipids. AGXX® has self-regenerating activity based on two coupled redox reactions taking place on the micro-galvanic silver and ruthenium elements on the surface of the material. They result in effective regeneration of the coating (Clauss-Lendzian et al., 2018).

In this study, we investigated the long-term antimicrobial effect of two different antimicrobial coatings. Three sets of V2A steel samples (uncoated, silver-coated, AGXX®-coated) were exposed and analyzed after six, 12, and 19 months on the ISS. Seventy-eight human pathogenic bacteria, which survived on the antimicrobial coatings or on the uncoated steel carrier (control) were phylogenetically affiliated and further characterized. The number of human pathogenic isolates decreased from V2A steel (*n* = 39) to V2A-Ag (*n* = 31) to V2A-AGXX® (*n* = 8). After 6 months of exposure, no bacteria survived on AGXX®, whereas six human pathogens were obtained after 12 and two after 19 months. From all materials, predominantly staphylococci and bacilli were isolated. Multi-antibiotic resistant, plasmid harboring staphylococcal and enterococcal ISS isolates transferred erythromycin, gentamicin and tetracycline resistance with average transfer frequencies of 10^−5^ transconjugants/recipient.

## Materials and Methods

### Preparation of Antimicrobial Metal Sheets

The material was provided by Largentec GmbH, Berlin, Germany. V2A (DIN ISO 1.4301) stainless steel sheets were used as reference material and as base material for Ag and AGXX® coatings. The coatings were prepared as described in detail in Clauss-Lendzian et al. (2018). Prior to use in the experiments, the metal sheets (coated and uncoated) were autoclaved at 121°C for 20 min. The metal sheets had a size of 4 cm^2^ each and were placed on the door to the bathroom of the ISS. Three sets of test sheets, one for each time point, - time points 12 and 19 months thus representing a cumulative bacterial load—were exposed on the ISS.

### Reference Strains

Bacterial strains used as reference in biofilm formation assays and PCRs or as recipients in mating experiments are listed in Table 1. *Staphylococcus* and *Enterococcus* strains were grown in Tryptic Soy Broth (TSB, Sigma-Aldrich Chemie GmbH, Munich, Germany) or Brain Heart Infusion broth (Carl Roth GmbH & Co. KG, Karlsruhe, Germany) at 37°C with shaking. *Bacillus* strains were grown in Lysogeny Broth (LB, Carl Roth GmbH & Co. KG, Karlsruhe, Germany) at 30°C with shaking.

**Table 1:** Bacterial species used as references for AB-R-screening, biofilm formation, plasmid isolation, and in biparental mating.

**Species**	**Genotype/Characteristics**	**References**
***Bacillus subtilis***		
BD662	pBD90 [*ermD*]; *trpC2, thr-5*	Schiwon et al., 2013
BD1156	pBD370 [*ermG*]; *leu, mer, hisH*	Schiwon et al., 2013
*Enterobacter cloacae* DSM46348	*ampC*	Schiwon et al., 2013
*Enterococcus casseliflavus* UC73	*aph(2)-Id, vanC*	Schiwon et al., 2013
*Enterococcus gallinarum* SF9117	*aph(2)-Ic, vanC, ermB*	Schiwon et al., 2013
***Enterococcus faecalis***		
DS16	*tetM*; pAD1, pAD2	Schiwon et al., 2013
RE25	pRE25 [*ermB*, cat_pIP501_, *aph(3)-III, sat4, ant(6)-Ia, tra^+^*], *tetM*	Schiwon et al., 2013
JH2-2	pIP501 [*cat*_pIP501_, *ermB, tra^+^*]	Schiwon et al., 2013
T9	clinical isolate, Tet^R^, strong biofilm former	Creti et al., 2006
OG1X	Strep^R^, Gent^R^, protease-negative	Schiwon et al., 2013
MISSEX 78	*pre*_pSK41_, *aph3-III, ermB, ermD, tetM, cat*_pIP501_	
TU-79	*aph3-III, ermB, tetM, tetO, cat*_pIP501_, *pre*_pSK41_	Schiwon et al., 2013
***Enterococcus faecium***		
SF11770	*aac(6)-Im, aph(2)-Ib, aac(6)-Ii, ant(4)-Ia, ant(6)-Ia, aph(3)-III, ermB, sat4, tetL, tetM, vanA, vanZ*	Schiwon et al., 2013
***Escherichia coli***		
PS84	*qnrS, sul2*	Broszat et al., 2014
Hm06-20	*qnrA, sul1*	Broszat et al., 2014
*Lactococcus lactis* K214	pK214 [*tetS, catLM, mdt(A), str, mob^+^*]	DSMZ
***Klebsiella pneumoniae***		
K2-78	*qnrB*	Broszat et al., 2014
DSM 16609	*blaSHV-5*	Broszat et al., 2014
***Staphylococcus aureus***		
SK5428	pSK41 [*ant(4)-Ia (synonym: aadD), aac(6)-Ie-aph(2)-Ia, ble, qacC, tra^+^*]	Schiwon et al., 2013
DSM13661	*mecA*	Schiwon et al., 2013
04-02981	strong biofilm former, methicillin resistant, *ermA*	Nuebel et al., 2010
*Staphylococcus epidermidis* MISSEX 66	*pre*_pSK41_, *aph3-III*	Schiwon et al., 2013
*Staphylococcus haemolyticus* VPS617	*tetK, mph(C), ermC, msr, blaZ, mecA, dfrA, aph(3)-III, aph(2)-Ia, aac(6)-Ie, ant(6)-IaInorA, sat4*	Schiwon et al., 2013

*DSMZ, German collection of microorganisms and cell cultures, Braunschweig. AB-R, antibiotic resistance; Strep^R^, streptomycin resistance; Tet^R^, tetracycline resistance; Gent^R^, gentamicin resistance; AB-R genes for resistance against ampicillin, ampC (E. cloacae DSM46348), ciprofloxacin, qnrA (E. coli Hm06-20), qnrB (K. pneumoniae K2-78), qnrS (E. coli PS84); erythromycin, ermA (S. aureus 04-02981), ermB (E. faecium SF11770), ermC (S. haemolyticus VPS617), ermD (B. subtilis BD662), ermG (B. subtilis BD1156); gentamicin, aac(6′)-Ie-aph(2′)-Ia (E. faecium SF11770), aph(2′)-ib (E. faecium SF11770), aph(2′)-ic (E. gallinarum SF9117), aph(2′)-id (E. casseliflavus UC73); kanamycin, aadD [S. aureus SK5428 (pSK41)], aph(3′)-III (E. faecalis RE25) against oxacillin, mecA (S. aureus DSM13661); ß-lactams, blaSHV-5 (K. pneumoniae DSM13661), blaZ (S. haemolyticus VPS617); sulfamethoxazole, sul1 (E. coli Hm06-20), sul2 (E. coli PS84), and tetracycline, tetK (S. haemolyticus VPS617), tetL (E. faecium SF11770), tetM (E. faecium SF11770), tetO (E. faecalis TU-79), tetS (L. lactis K214)*.

### Bacteria Isolation and Phylogenetic Affiliation

Bacteria were isolated from V2A steel surfaces (uncoated, Ag-coated, AGXX®-coated) exposed on the ISS for 6, 12 and 19 months, respectively. The bacteria were detached from the surfaces by rinsing with Phosphate Buffered Saline (PBS) followed by cultivation in Reasoner's 2A broth (R2A, Lab M Limited, Heywood, England) at 25° and 37°C under shaking. Appropriate dilutions of the cultures were passaged several times onto R2A agar until pure isolates were obtained. Isolates were phylogenetically affiliated by matrix-assisted laser desorption ionization time-of-flight mass spectrometry (MALDI-TOF MS, Bruker Daltonics MALDI Biotyper system) according to the manufacturer's instructions (Bruker Daltonics). Mass spectra were compared with the MALDI-BDAL Database (Version 3.1, 7311rntries). If identification with MALDI-TOF MS failed, the isolate was sent for 16S rRNA gene sequencing (SMB Ruedersdorf, Germany). Analysis of the 16S rDNA sequences was performed with BLAST (http://blast.ncbi.nlm.nih.gov/Blast.cgi?PROGRAM=blastn&PAGE_TYPE=BlastSearch&LINK_LOC=blasthome) and ChromasPro (Version 2.1.8). The isolates are denominated according to following scheme (i) the material they were isolated from, (ii) the exposure time on the ISS in months, and (iii) the order of isolation, e.g., *E. faecalis* V2A-12-03 was isolated from uncoated V2A steel after 12 months exposure, and it is the third isolate obtained from this material at this time-point.

### Biofilm Screening Assay

Biofilm formation test was carried out according to Vaishampayan et al. (2018). *E. faecalis* T9 and *S. aureus* 04-02981, both strong biofilm formers, were used as positive controls (Schiwon et al., 2013; Vaishampayan et al., 2018). For *Staphylococcus* spp., TSB, for *E. faecalis*, BHI medium was used as negative control (Schiwon et al., 2013). Biofilm formation was measured in EnSpire Multimode Plate Reader 2300-0000 (Perkin Elmer, Turku, Finland) at 570 nm (OD_570_). The assays were performed in triplicates. Normalized biofilm formation was calculated by dividing the biofilm measure at OD_570_ by the bacterial growth at OD_600_. Biofilm classification criteria were applied according to Nyenje et al. (2013).

### Antibiotic Disc Diffusion Method

Antibiotic resistance of the isolates toward 15 different antibiotics was analyzed with the disc diffusion method (discs from Oxoid, Wesel, Germany) on Mueller Hinton agar (Sifin diagnostic GmbH, Berlin, Germany) according to the guidelines of the Clinical and Laboratory Standards Institute, (CLSI, 2013). Details are given in Table 2. Each test was performed in triplicates. For sulfamethoxazole (RL25), no comparable data were found for Staphylococci, Enterococci and Bacilli. Thus, isolates lacking an inhibition zone were classified as resistant, those without inhibition zone were classified as susceptible.

**Table 2:** Antibiotic disc diffusion method.

			**References**		
**Antibiotic**	**Abbreviation**	**Concentration μg/mL**	***Staphylococcus* spp**.	***Enterococcus* spp**.	***Bacillus* spp**.
Ampicillin	AMP	10	CLSI, 2013	EUCAST, 2013	Mohammadou et al., 2014
Chloramphenicol	C	30	EUCAST, 2013	Liofilchem®, 2017^a^	Mohammadou et al., 2014
Ciprofloxacin	CIP	5	EUCAST, 2013	EUCAST, 2013	Banerjee et al., 2011
Gentamicin	CN	10	EUCAST, 2013	Oliveira et al., 2010	Banerjee et al., 2011
Clindamycin	DA	10	Liofilchem®, 2017^a^	Liofilchem®, 2017^a^	Liofilchem®, 2017^a^
Doxycycline	DO	30	Liofilchem®, 2017^a^	Liofilchem®, 2017^a^	Liofilchem®, 2017^a^
Erythromycin	E	15	Liofilchem®, 2017^a^	Liofilchem®, 2017^a^	Mohammadou et al., 2014
Kanamycin	K	4	Liofilchem®, 2017^a^	Liofilchem®, 2017^a^	Liofilchem®, 2017^a^
Cephalothin	KF	30	Liofilchem®, 2017^a^	Liofilchem®, 2017^a^	Liofilchem®, 2017^a^
Meropenem	MEM	10	Liofilchem®, 2017^a^	Andrews, 2007	Liofilchem®, 2017^a^
Oxacillin	OX	5	Liofilchem®, 2017^a^	Liofilchem®, 2017^a^	Liofilchem®, 2017^a^
Tigecycline	TCG	5	EUCAST, 2013	EUCAST, 2013	EUCAST, 2013
Tetracycline	TE	10	EUCAST, 2013	Andrews, 2007	EUCAST, 2013
Vancomycin	VA	30	EUCAST, 2013	Tamanna et al., 2014	EUCAST, 2013

*For Enterococcus spp., inhibition zones from DA10, E5, K5, KF30, OX5 and for Bacillus spp., inhibition zones from DA10, DO30, K5, KF30, MEM10, OX5, TCG15, TE10 were evaluated as for Staphylococcus spp*.

a *http://www.liofilchem.net/antibioticdisc/*.

### PCR Assays

For the PCR assays, cell lysates prepared from 100 μL overnight cultures were used. Cell pellets were re-suspended in 20 μL lysis buffer (50 mM NaOH, 0.25% sodium dodecyl sulfate) and incubated at 95°C for 20 min. Prior to use in PCR, they were diluted 1:10 with distilled water. Twenty-five microliter PCR reactions contained 0.125 μL Taq-Polymerase (5 U/μL), 2.5 μL 1x PCR buffer, 0.2 μM of each primer (Table 3), 0.5 μL of deoxynucleoside triphosphates (200 μM) and 1 μL template DNA (lysate). DNA amplifications were carried out in a Biometra T3 Thermocycler (Analytik Jena AG, Jena, Germany). The temperature profiles are given in Supplementary Table 1.

**Table 3:** Oligonucleotides used for the detection of antibiotic resistance genes.

**Primer**	**Antibiotic**	**Sequence (5′ → 3′)**	**GenBank**	**Amplicon size [bp]**	**Annealing**	**References**
			**Acc. No**.		**temperature**	
					**[^◦^C]**	
aac6-aph2a fw	gentamicin	GCCAGAACATGAATTACACGAG	NC_005024	610	56	Schiwon et al., 2013
aac6-aph2a rev		CTGTTGTTGCATTTAGTCTTTCC				
aadD_pSK41 fw	kanamycin	TGTCGTTCTGTCCACTCCTG	AF051917	525	62	Schiwon et al., 2013
aadD_pSK41 rev		ATGAATGGACAACCGGTGAG				
ampC fw	ampicillin	GTGACCAGATACTGGCCACA	AJ005633	821	55	Schiwon et al., 2013
ampC rev		TTACTGTAGCGCCTCGAGGA				
aph(2)-Ib fw	gentamicin	AGGATGCCCTTGCATATGATGAAGCGACGT	AF207840	449	56	Schiwon et al., 2013
aph(2)-Ib rev		ATCAGCATAAGGCGCCGGAAGTAGCAGAAA				
aph(2)-Ic fw	gentamicin	AGCATACAATCCGTCGAGTCGCTTGGTGAG	U51479	641	56	Schiwon et al., 2013
aph(2)-Ic rev		CTGGCGCTGCAACTTGCTGAGTTCATGAAT				
aph(2)-Id fw	gentamicin	GTGGTTTTTACAGGAATGCCATC	AF016483	134	56	Schiwon et al., 2013
aph(2)-Id rev		CCCTCTTCATACCAATCCATATAACC				
aph3-III fw	kanamycin	CCGCTGCGTAAAAGATAC	X92945	592	56	Schiwon et al., 2013
aph3-III rev		GTCATACCACTTGTCCGC				
blaSHV-5 fw	ß-lactams	TGTTAGCCACCCTGCCGCT		825	60	Schiwon et al., 2013
blaSHV-5 rev		GTTGCCAGTGCTCGATCAG				
blaZ fw	ß-lactams	TTAAAGTCTTAC CGAAAGCAG	AB245468	777	60	Sidhu et al., 2002
blaZ rev		TAAGAGATTTGC CTATGCTT				
ermA fw	erythromycin	ACGATATTCACGGTTTACCCACTTA	WP_001072201	584	58	Khan et al., 1999
ermA rev		AACCAGAAAAACCCTAAAGACACG				
ermB fw	erythromycin	GCATTTAACGACGAAACTGGCT	U00453	572	56	Schiwon et al., 2013
ermB rev		GACAATACTTGCTCATAAGTAATGGT				
ermC fw	erythromycin	CGTAACTGCCATTGAAATAGACC	V01278	519	58	Schiwon et al., 2013
ermC rev		TCCTGCATGTTTTAAGGAATTG				
ermD fw	erythromycin	CGGGCAAATATTAGCATAGACG	M29832	463	56	Schiwon et al., 2013
ermD rev		ATTCTGACCATTGCCGAGTC				
ermG fw	erythromycin	TGCAGGGAAAGGTCATTTTAC	M15332	483	56	Schiwon et al., 2013
ermG rev		AACCCATTTCATTACAAAAGTTTC				
mecA fw	methicillin	TAATAGTTGTAGTTGTCGGGTTTG	X52593	707	60	Schiwon et al., 2013
mecA rev		TAACCTAATAGATGTGAAGTCGCT				
qnrB (B1, B7) fw	fluoroquinolone	AGCGGCACTGAATTTAT		497	56	Broszat, 2014
qnrB (B1, B7) rev		GTTTGCTGCTCGCCAGTC				
qnrS1 fw	fluoroquinolone	GGAAACCTACAATCATACATA		600	56	Broszat, 2014
qnrS1 rev		GTCAGGATAAACAATACC				
sul1 fw	sulfamethoxazole	CACCGGAAACATCGCTGCA		158	60	Broszat, 2014
sul1 rev		AAGTTCCGCCGCAAGGCT				
sul2 fw	sulfamethoxazole	CTCCGATGGAGGCCGGTAT		190	60	Broszat, 2014
sul2 rev		GGGAATGCCATCTGCCTTGA				
tetK_pT181 fw	tetracycline	TTTGAGCTGTCTTGGTTCATTG	CP000045	539	55	Schiwon et al., 2013
tetK_pT181 rev		AGCCCACCAGAAAACAAACC				
tetL	tetracycline	CATTTGGTCTTATTGGATCG	AY081910	475	55	Schiwon et al., 2013
tetL		ATTACACTTCCGATTTCGG				
tetM fw	tetracycline	GAACTCGAACAAGAGGAAAGC	M85225	729	55	Schiwon et al., 2013
tetM rev		ATGGAAGCCCAGAAAGGAT				
tetO fw	tetracycline	GGATGGCATACAGGCACAGA	M18896	737	55	Schiwon et al., 2013
tetO rev		GTTTGGATCATAGGGAGAGGAT				
tetS fw	tetracycline	TGGTCAACGGCTTGTCTATG	X92946	546	55	Schiwon et al., 2013
tetS rev		AGCCCAGAAAGGATTTGGAG				

*Reference strain for bla-SHV-5, K. pneumoniae DSM13661; sul1, E. coli Hm06-20; sul2, E. coli PS84*.

### Plasmid DNA Isolation

Plasmid DNA from Staphylococci was extracted as described in Schiwon et al. (2013) with some minor modifications. After washing the plasmid DNA with 70% ethanol, 1 μL of RNase A (10 μg/mL; Merck KGaA, Darmstadt) and 3 μL of Proteinase K (20 mg/mL; Merck KGaA, Darmstadt) were added, followed by 1 h incubation at room temperature. Plasmid DNA extraction from Enterococci was performed as described in (Schiwon et al., 2013).

### Mating Assays

On basis of multiple antibiotic resistance and occurrence of plasmids >20 kbp, ISS isolates were selected as donors for biparental matings. As recipients, the methicillin resistant clinical isolate, *S. aureus* 04-02981 and the *E. faecalis* lab strain OG1X were selected. Details on all of the matings are given in Table 4. Overnight cultures of Staphylococci were diluted 1:5 in TSB medium, overnight cultures of Enterococci 1:5 in BHI medium containing the appropriate antibiotics (Table 4) and grown until OD_600_ = 0.5. Donors and recipients were washed with PBS prior to mixing in 1:10 ratio, spotted onto a TSA plate for *Staphylococcus* recipients, on a BHI plate for *Enterococcus* recipients and incubated for 16 h at 37°C. Cells were recovered in 1 mL PBS, serial dilutions were incubated at 37°C on TSA/BHI plates for 16 h to enumerate transconjugants. The number of recipients was also determined after 16 h at 37°C. Transfer frequencies are given as number of transconjugants/recipient.

**Table 4:** Efficiency of gentamicin, erythromycin, and tetracycline resistance transfer from ISS-isolates to *S. aureus* 04-02981 and *E. faecalis* OG1X.

**Donor**	**Selection**	**Recipient**	**Selection**	**Transconjugant selection**	**Transfer efficiency per recipient**
*E. faecalis* V2A-12-03	CN10	*S. aureus* 04-02981	CIP5	CN10, CIP5	8.3 × 10^−4^
*E. faecalis* V2A-AGXX-12-02	CN10		CIP5	CN10, CIP5	-
*E. faecalis* V2A-AGXX-12-03	CN10		CIP5	CN10, CIP5	9.2 × 10^−7^
*S. hominis* V2A-6-05	TE10		CIP5	TE10, CIP5	2.7 × 10^−5^
*S. hominis* V2A-6-06	TE10		CIP5	TE10, CIP5	3.3 × 10^−8^
*S. haemolyticus* V2A-12-08	TE10		CIP5	TE10, CIP5	6.6 × 10^−7^
*S. hominis* V2A-AG-12-06	TE10		CIP5	TE10, CIP5	4.2 × 10^−4^
*S. hominis* V2A-AGXX-12-01	TE10		CIP5	TE10, CIP5	6.8 × 10^−4^
*S. hominis* V2A-AGXX-12-03	TE10		CIP5	TE10, CIP5	-
*S. haemolyticus* V2A-AGXX-12-05	TE10		CIP5	TE10, CIP5	1.2 × 10^−7^
*S. hominis* V2A-6-03	E15	*E. faecalis* OG1X	SM1000	E15, SM1000	1.6 × 10^−4^
*S. hominis* V2A-6-11	E15		SM1000	E15, SM1000	4.2 × 10^−4^
*S. aureus* V2A-6-13	E15		SM1000	E15, SM1000	9.1 × 10^−5^
*S. aureus* V2A-6-14	E15		SM1000	E15, SM1000	2.5 × 10^−4^
*E. faecali*s V2A-12-03	E15		SM1000	E15, SM1000	-
*S. haemolyticus* V2A-AGXX-12-05	E15		SM1000	E15, SM1000	-
*S. hominis* V2A-6-05	E15		CN10	E15, CN10	-
*S. hominis* V2A-6-06	E15		CN10	E15, CN10	7.9 × 10^−6^
*S. hominis* V2A-6-09	E15		CN10	E15, CN10	-
*S. hominis* V2A-6-10	E15		CN10	E15, CN10	-
*S. hominis* V2A-6-12	E15		CN10	E15, CN10	-
*S. hominis* V2A-12-04	E15		CN10	E15, CN10	1.6 × 10^−6^
*S. haemolyticus* V2A-12-08	E15		CN10	E15, CN10	1.1 × 10^−6^
*S. hominis* V2A-AG-12-05	E15		CN10	E15, CN10	-
*S. hominis* V2A-AG-12-06	E15		CN10	E15, CN10	5.1 × 10^−6^
*S. hominis* V2A-AG-12-08	E15		CN10	E15, CN10	-
*E. faecalis* V2A-AGXX-12-03	E15		CN10	E15, CN10	-
*S. hominis* V2A-AGXX-12-06	E15		CN10	E15, CN10	1.1 × 10^−5^

*CIP, Ciprofloxacin; CN, gentamicin; E, erythromycin; SM, streptomycin; TE, tetracycline. (-) no transconjugants were obtained. The concentrations of the antibiotics are given in μg/mL*.

## Results

### Bacterial Isolates From V2A, V2A-Ag and V2A-AGXX® Surfaces

A total number of 112 bacterial isolates were recovered from the different materials after the three time intervals (6, 12, and 19 months). 73.6% of the isolates are human pathogens. All isolates were identified to species level by MALDI-TOF biotyping or 16S rRNA gene sequencing. In total, 49 isolates were obtained after 6 months, 51 after 12 months and 22 after 19 months exposure of the antimicrobial materials on the ISS. The non-human pathogenic bacteria include *Bacillus* spp. (*n* = 20; *B. astrophaeus, B. infantis, B. korlensis, B. licheniformis, B. megaterium, B. niacini, B. pumilus, B. tequilensis*, and *B. thuringiensis*), *Enhydrobacter aerosaccus* (*n* = 2), *Micrococcus yunannensis* (*n* = 1), *Paenibacillus polymyxa* (*n* = 1), *Pseudomonas psychrotolerans* (*n* = 1), and *Staphylococcus capitis* (*n* = 9). To assess the infection risk for the crew, only the human-pathogenic bacteria (*n* = 78) were characterized in terms of biofilm formation and antibiotic resistance profile. Three *Moraxella osloensis* strains obtained from V2A (*n* = 1) and V2A-Ag (*n* = 2) after 19 months were the only Gram-negative human-pathogenic bacteria. Seventy-five Gram-positive human pathogenic bacteria were selected for the study: 32 from 6 months, 21 from 12 months, and 22 isolates from 19 months exposure.

**Figure 1:**
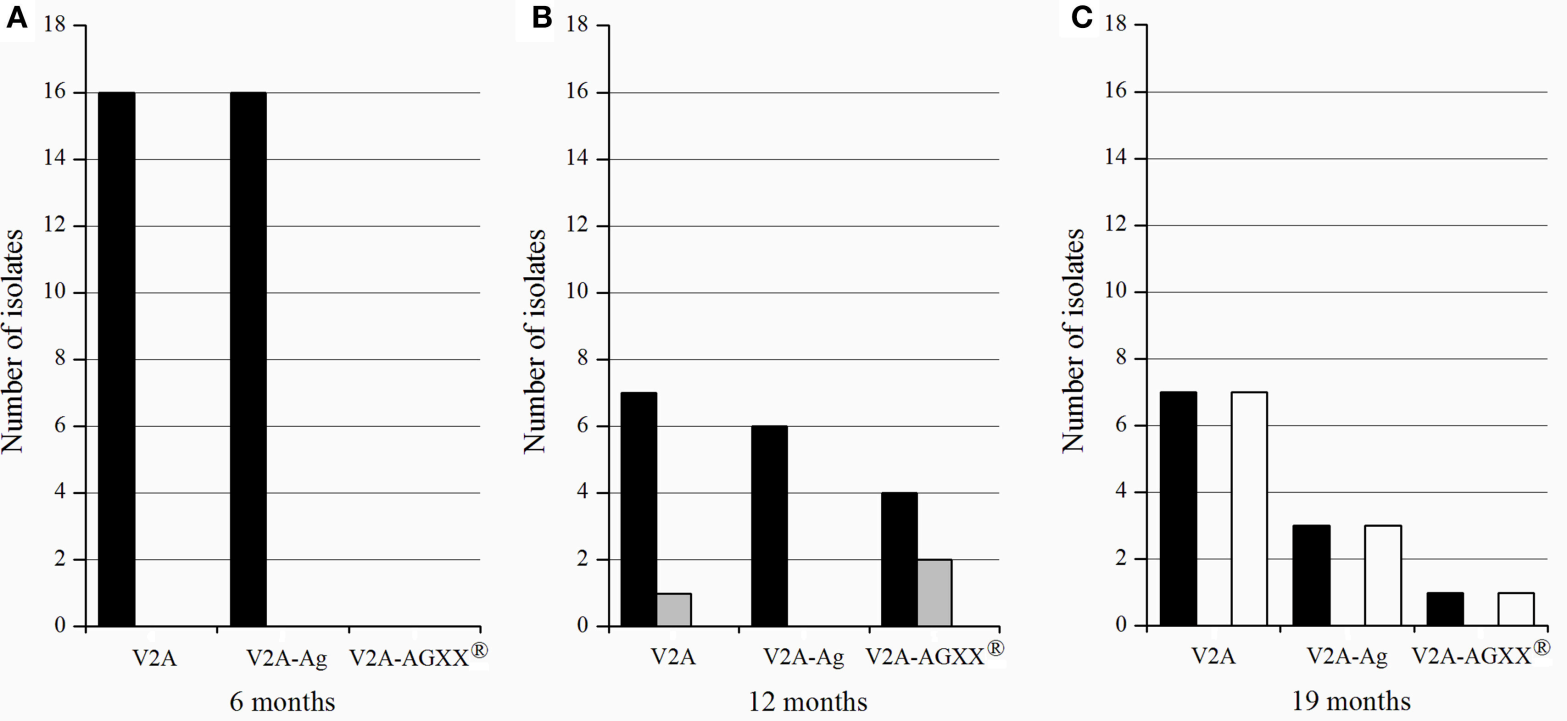
Number of Gram-positive pathogenic bacteria recovered from the different materials (V2A, V2A-Ag, V2A-AGXX®), after 6 months **(A)**, 12 months **(B)**, and 19 months **(C)** exposure on the ISS. In black, *Staphylococcus* spp.; gray, *E. faecalis*; white, *B. cereus*.

The longer the exposure time of the three materials, the higher was the bacterial diversity on the materials (Figure 1 and Table 5). All pathogenic isolates recovered from V2A and V2A-Ag after six months belonged to the genus *Staphylococcus*. No bacteria were recovered from AGXX® after 6 months. In total, 17 Staphylococci and three *E. faecalis* were detected after 12 months: Seven Staphylococci and one *E. faecalis* strain from V2A, six Staphylococci from V2A-Ag and four Staphylococci and two *E. faecalis* strains from AGXX®. After 19 months, seven Staphylococci and seven *B. cereus* strains were recovered from V2A and three Staphylococci and three *B. cereus* strains from V2A-Ag. Only one *B. cereus* and one *S. epidermidis* strain were isolated from AGXX® after 19 months exposure. In summary, a considerably lower bacterial number survived on AGXX® than on the other two surfaces. Nevertheless, the silver coating also showed a slight antimicrobial effect.

**Table 5:** Characteristics of all isolates from V2A, V2A-Ag, V2A-AGXX® after 6, 12, and 19 months.

**No**.	**Name**	**Species**	**Biofilm formation**	**AB-R**		**Number of plasmid-bands >20 kbp**
				**Phenotype**	**Genotype**	
1	V2A-6-01	*S. hominis*	+++	AMP10, DA10, E15, RL25	n.d.	n.d.
2	V2A-6-02	*S. hominis*	++	DA10, E15, RL25	*ermC*	n.d.
3	V2A-6-03	*S. hominis*	+++	AMP10, DA10, E15, RL25	*ermC*	1
4	V2A-6-04	*S. hominis*	+++	DA10, E15, RL25	n.d.	n.d.
5	V2A-6-05	*S. hominis*	+++	AMP10, E15, RL25, TE10	*tetK, ermC*	2
6	V2A-6-06	*S. hominis*	++	AMP10, E15, RL25, TE10	*tetK, tetO, ermC*	2
7	V2A-6-07	*S. hominis*	++	AMP10, E15, RL25, TE10	*tetK, ermC*	1
8	V2A-6-08	*S. hominis*	++	DA10, E15, RL25	n.d.	n.d.
9	V2A-6-09	*S. hominis*	+++	AMP10, DA10, E15, RL25	*tetK*	2
10	V2A-6-10	*S. hominis*	+++	AMP10, E15, RL25, TE10	n.d.	0
11	V2A-6-11	*S. hominis*	+++	AMP10, E15, RL25, TE10	*ermC*	1
12	V2A-6-12	*S. hominis*	+++	AMP10, E15, RL25, TE10	*ermC*	1
13	V2A-6-13	*S. aureus*	+++	AMP10, E15, K5, RL25	*ermC*	1
14	V2A-6-14	*S. aureus*	+++	AMP10, DA10, E15, K5, RL25	*tetK*	0
15	V2A-6-15	*S. hominis*	+++	AMP10, E15, RL25, TE10	*ermC*	1
16	V2A-6-16	*S. aureus*	++	AMP10, E15, K5, RL25, TE10	*ermC*	n.d.
17	V2A-AG-6-01	*S. epidermidis*	+++	E15, RL25	*ermC*	n.d.
18	V2A-AG-6-02	*S. epidermidis*	+++	E15, RL25	n.d.	n.d.
19	V2A-AG-6-03	*S. aureus*	+++	AMP10, E15, RL25	*ermC*	n.d.
20	V2A-AG-6-04	*S. aureus*	+++	AMP10, E15, RL25	*ermC*	n.d.
21	V2A-AG-6-05	*S. aureus*	+++	AMP10, E15, K5, RL25	n.d.	n.d.
22	V2A-AG-6-06	*S. epidermidis*	+++	E15, RL25	n.d.	n.d.
23	V2A-AG-6-07	*S. aureus*	++	AMP10, E15, RL25	n.d.	n.d.
24	V2A-AG-6-09	*S. epidermidis*	+++	E15, RL25	n.d.	n.d.
25	V2A-AG-6-11	*S. aureus*	+++	AMP10, E15, RL25	n.d.	n.d.
26	V2A-AG-6-14	*S. aureus*	+++	AMP10, E15, RL25	n.d.	n.d.
27	V2A-AG-6-15	*S. epidermidis*	+++	AMP10, E15, RL25	n.d.	n.d.
28	V2A-AG-6-16	*S. epidermidis*	+++	E15, RL25	*ermC*	n.d.
29	V2A-AG-6-21	*S. epidermidis*	+++	E15, RL25	n.d.	n.d.
30	V2A-AG-6-22	*S. epidermidis*	+++	E15, RL25	n.d.	n.d.
31	V2A-AG-6-23	*S. epidermidis*	+++	AMP10, E15, RL25	n.d.	n.d.
32	V2A-AG-6-24	*S. epidermidis*	+++	E15, RL25	n.d.	n.d.
33	V2A-12-02	*S. hominis*	++	AMP10, E15, MEM10, RL25	n.d.	n.d.
34	V2A-12-03	*E. faecalis*	+++	C30, CN10, DA10, DO30, E15, K5, MEM10, RL25, TE10	*aac6-aph2a, aph3-III, ermC*	1
35	V2A-12-04	*S. hominis*	+++	AMP10, E15, K5, RL25, TE10	*aph3-III, tetO*	1
36	V2A-AGXX-12-01	*S. hominis*	+++	AMP10, E15, RL25, TE10	*tetK, tetL*	1
37	V2A-AG-12-03	*S. hominis*	+++	AMP10, K5, RL25	*aadD, aph3-III*	1
38	V2A-AG-12-04	*S. hominis*	+++	AMP10	n.d.	n.d.
39	V2A-AG-12-05	*S. hominis*	+++	AMP10, E15, K5, MEM10, RL25	*aph3-III, aadD, ermC*	0
40	V2A-AGXX-12-02	*E. faecalis*	+++	CN10, DO30, K5, RL25	*aph(2)-ic, aph3-III, aadD*	1
41	V2A-AGXX-12-03	*E. faecalis*	+++	CN10, DA10, E15, K5, RL25, TE10	*aph(2)-ic, aadD, aph3-III, ermB, tetK, tetM, tetO*	1
42	V2A-AG-12-06	*S. hominis*	+++	AMP10, DO30, E15, RL25, TCG15, TE10	*tetK*	1
43	V2A-12-07	*S. epidermidis*	+++	AMP10, DO30, RL25	n.d.	n.d.
44	V2A-AGXX-12-05	*S. haemolyticus*	+++	C30, DO30, E15, RL25, TE10	*tetO*	3
45	V2A-AG-12-08	*S. hominis*	+++	AMP10, DA10, E15, RL25	*ermC*	2
46	V2A-12-08	*S. haemolyticus*	+++	C30, DO30, E15, RL25, TE10	*tetK*	1
47	V2A-AG-12-10	*S. hominis*	+++	RL25	n.d.	n.d.
48	V2A-12-09	*S. lugdunensis*	+++	RL25	n.d.	n.d.
49	V2A-AGXX-12-06	*S. hominis*	+++	AMP10, DA10, E15, RL25	*ermC*	1
50	V2A-AGXX-12-09	*S. hominis*	+++	AMP10, RL25	n.d.	n.d.
51	V2A-12-10	*S. caprae*	+++	AMP10, C30, E15, RL25	*ermC*	n.d.
52	V2A-12-12	*S. lugdunensis*	+++	RL25	0	0
53	V2A-12-13	*S. hominis*	+++	AMP10, DA10, RL25	n.d.	n.d.
54	V2A-19-02	*S. hominis*	++	AMP10, E15, RL25	n.d.	n.d.
55	V2A-AGXX-19-01	*S. epidermidis*	+	E15, RL25	n.d.	n.d.
56	V2A-19-03	*S. hominis*	+++	E15, RL25	n.d.	n.d.
60	V2A-19-05	*S. hominis*	+++	E15, RL25	n.d.	n.d.
61	V2A-19-06	*S. hominis*	+++	E15, RL25	n.d.	n.d.
62	V2A-19-07	*S. hominis*	+++	E15, RL25	n.d.	n.d.
63	V2A-AG-19-06	*S. hominis*	+++	RL25	n.d.	n.d.
64	V2A-19-09	*S. hominis*	+++	E15, RL25	n.d.	n.d.
65	V2A-19-10	*S. hominis*	+++	E15, RL25	n.d.	n.d.
66	V2A-AG-19-07	*S. hominis*	+++	E15, RL25	n.d.	n.d.
67	V2A-AG-19-08	*S. hominis*	+++	E15, RL25	n.d.	n.d.
68	V2A-19-14	*B. cereus*	+++	AMP10, K5, OX5, RL25	n.d.	n.d.
69	V2A-19-15	*B. cereus*	+	AMP10, K5, OX5, RL25	n.d.	n.d.
70	V2A-AG-19-10	*B. cereus*	+	AMP10, C30, E15, K5, OX5, RL25	n.d.	n.d.
71	V2A-19-16	*B. cereus*	+	AMP10, OX5, RL25, TCG15	n.d.	n.d.
72	V2A-19-17	*B. cereus*	++	AMP10, K5, OX5, RL25	n.d.	n.d.
73	V2A-19-18	*B. cereus*	+	AMP10, OX5	n.d.	n.d.
74	V2A-AG-19-12	*B. cereus*	+	AMP10, OX5, RL25	n.d.	n.d.
75	V2A-19-19	*B. cereus*	++	AMP10, OX5, RL25	n.d.	n.d.
76	V2A-19-20	*B. cereus*	++	AMP10, OX5, RL25	n.d.	n.d.
77	V2A-AG-19-14	*B. cereus*	+	AMP10, E15, OX5, RL25	n.d.	n.d.
78	V2A-AGXX-19-03	*B. cereus*	+	AMP10, OX5, RL25	n.d.	n.d.

*AB-R, antibiotic resistance; +++, strong biofilm formation; ++, moderate biofilm formation; +, weak biofilm formation; AMP, ampicillin; C, chloramphenicol; CN, gentamicin; DA, clindamycin; DO, doxycycline; E, erythromycin; K, kanamycin; MEM, meropenem; OX, oxacillin; RL, sulfamethoxazole; TCG, tigecycline; TE, tetracycline. The concentration of the antibiotics is given in μg/mL. ermC, ermB, erythromycin resistance genes; aac6-aph2a, aph(2)-ic, gentamicin resistance genes; blaSHV-5, ß-lactam antibiotic resistance gene; aadD, aph3-III, kanamycin resistance genes; tetK, tetM, tetL, tetO, tetracycline resistance genes. n.d., not determined*.

### Biofilm Formation of Pathogenic ISS-Isolates

Biofilm formation of pathogenic isolates was determined by crystal violet staining, biofilms were classified according to Nyenje et al. (2013). The data are summarized in Table 5. Twenty-six V2A-isolates showed strong (66.7% of all pathogenic isolates from V2A-steel), ten moderate (25.5%) and three weak (7.8%) biofilm formation. Twenty-one isolates from V2A-Ag were strong biofilm formers (91.3% of all pathogenic isolates from V2-Ag), one isolate showed moderate (4.3%), one isolate weak (4.3%) biofilm formation. Of the eight AGXX®-isolates, six had strong (75.0% of all pathogenic isolates from V2A-AGXX®) and two (25%) weak biofilm formation ability. Interestingly, 43 Staphylococci (52 pathogenic Staphylococci in total) formed strong biofilms (82.7%), eight Staphylococci (15.4%) were moderate biofilm formers and one *Staphylococcus* isolate (1.9%) formed only weak biofilms. Of the *B. cereus* isolates (11 in total), one showed strong (9.1%), three moderate (27.3%), and seven (63.6%) showed only weak biofilm formation capacity. In contrast, all three *E. faecalis* isolates were classified as strong biofilm formers.

### Prevalence of Antibiotic Resistances in the Pathogenic Isolates

Antibiotic sensitivity testing of the isolates showed that 32.0% of the pathogenic isolates were resistant to <3 of the tested antibiotics (15 antibiotics in total were tested), 68.0% were resistant to three or more antibiotics. Eighteen isolates had three antibiotic resistances (24.0% of the isolates), 23 isolates were resistant to four antibiotics (30.7% of the isolates), six isolates were resistant to five antibiotics (8.0%) and three isolates had six different antibiotic resistances (4.0%). *E. faecalis* V2A-12-03 (from V2A steel after 12 months) had the highest number of resistances. It was resistant to nine different antibiotics, chloramphenicol, gentamicin, clindamycin, doxycycline, erythromycin, kanamycin, meropenem, sulfamethoxazole, and tetracycline.

In total, 97.3% of the pathogenic Gram-positive isolates were resistant to 25 μg sulfamethoxazole, 74.7% were resistant to 15 μg erythromycin and 61.3% were resistant to 10 μg ampicillin. Interestingly, these resistances were found with similar prevalence on all three surfaces, irrespective of the exposure time. No oxacillin resistant *Staphylococcus* was detected, whereas all *B. cereus* isolates (all of the 11 isolates after 19 months) were resistant to oxacillin. One *B. cereus* (V2A-AG-19-10) isolate showed resistances against six different antibiotics (AMP10, C30, E15, K5, OX5, RL25).

None of the isolates was resistant to vancomycin or cephalothin. Two *E. faecalis* (V2A-AGXX-12-02,-03) isolates were resistant to six antibiotics (CN10, DA10, E15, K5, RL25, TE10) and one *E. faecalis* isolate (V2A-12-03) was resistant to nine antibiotics (C30, CN10, DA10, DO30, E15, K5, MEM10, RL25, TE10). Meropenem resistance was detected in three strains, *E. faecalis* V2A-12-03, *S. hominis* V2A-12-04, and *S. hominis* V2A-AG-12-05.

To identify the resistance genes in the isolates resistant to three or more antibiotics (68.0% of the pathogenic isolates), gene-specific PCRs were performed. Gentamicin [*aac6-aph2a* (*n* = 1)*, aph(2)-ic* (*n* = 2)], kanamycin [*aadD* (*n* = 4)*, aph3-III* (*n* = 5)], erythromycin [*ermC* (*n* = 19), *ermB* (*n* = 1)], and tetracycline [*tetK* (*n* = 9)*, tetL* (*n* = 1), t*etM* (*n* = 1)] resistance genes were detected in the number of isolates indicated in parentheses (Table 5). *ermC* and *tetK* were the most prevalent resistance genes. No sulfamethoxazole (*sul1, sul2*) resistance gene was found in any of the isolates.

### Plasmid Profiles of ISS-Isolates

Exemplarily, plasmid DNA profiles of 20 out of total 45 staphylococcal isolates resistant to three or more antibiotics forming moderate or strong biofilms were obtained (Table 5). All isolates contained plasmids <20 kbp, the number of plasmid bands varied from one to seven. Interestingly, 17 isolates harbored plasmids >20 kbp likely able to self-transfer. Plasmid DNA profiles were also obtained from the three *E. faecalis* isolates; all of them were multi-drug resistant and strong biofilm formers. All, *E. faecalis* V2A 12-03, *E. faecalis* V2A-AGXX-12-02 and *E. faecalis* V2A-AGXX-12-03 harbored putative conjugative plasmids >20 kbp. Interestingly, *E. faecalis* V2A 12-03 showed additionally three small plasmid bands in the size range between 3 and 1.5 kbp.

### Mating Experiments

Antibiotic resistance transfer of selected ISS-isolates was studied in biparental matings (Laverde et al., 2017). Isolates resistant to tetracycline, gentamicin or erythromycin and harboring a plasmid >20 kbp were selected as donors, plasmid-free *S. aureus* 04-02981 and *E. faecalis* OG1X were used as recipients. The results of all of the matings are summarized in Table 4. Gentamicin resistance transfer to *S. aureus* 04-02981 was successful from *E. faecalis* V2A-12-03 (*aac6-aph2a-*encoded gentamicin resistance) with a transfer frequency of 8.3 × 10^−4^ transconjugants/recipient and from *E. faecalis* V2A-AGXX-12-03 (*aph(2)-ic*-encoded gentamicin resistance) with a transfer frequency of 9.2 × 10^−7^ transconjugants/recipient.

Erythromycin resistance transfer of six *Staphylococcus* donors harboring the *ermC* resistance gene and of three *Staphylococcus* donors harboring an unknown erythromycin resistance gene to *E. faecalis* OG1X was successful with transfer frequencies in the range of 1.1 × 10^−6^ to 4.2 × 10^−4^ transconjugants/recipient. Tetracycline resistance transfer from four *S. hominis* strains and two *S. haemolyticus* strains to *S. aureus* 04-02981 was successful. Three of the staphylococci harbored only the *tetK* resistance gene, one only *tetO*. One *S. hominis* strain harbored *tetK* and *tetO*, while another harbored the resistance genes *tetK* and *tetL*. Tetracycline resistance transfer frequencies varied considerably ranging from 3.3 × 10^−8^ to 6.8 × 10^−4^ transconjugants/recipient.

Ten out of the 17 successful matings were randomly chosen for plasmid DNA isolation of the transconjugants. In nine of the ten matings large plasmid bands comparable in size to those of the donors were detected in the transconjugants (data not shown).

## Discussion

We proved that the novel antimicrobial coating AGXX® strongly reduced the bacterial load on surfaces on the ISS particularly prone to microbial contamination. However, over time—with exposure times >6 months—some nosocomial pathogens survived even on the novel antimicrobial coating. Moreover, an interesting shift in the composition of the microbial communities was observed over time.

### Bacterial Survivors Isolated From V2A, V2A-Ag and V2A-AGXX® Surfaces

The bacterial community isolated from the surfaces was always dominated by *Staphylococcus* spp. (63.4% of 112 isolates) and *Bacillus* spp. (24.1%) irrespective of the exposure time. 46.4% of the Staphylococci are affiliated to the coagulase-negative Staphylococci, including pathogens such as *S. epidermidis, S. lugdunensis, S. haemolyticus, S. hominis*, and *S. caprae*. Coagulase-positive Staphylococci such as *S. aureus* (8.9% of all isolates) were only found on V2A and V2A-Ag surfaces after 6 months exposure. *B. cereus* (9.8% of all isolates) was the only pathogenic *Bacillus*. Only three *E. faecalis* (2.7% of all isolates) were recovered from V2A and V2A-AGXX® surfaces after 12 months. Schiwon et al. reported that predominantly *S. hominis, S. aureus*, and *S. epidermidis* were detected on crew-members and in air-filters on the ISS (Schiwon et al., 2013). *S. hominis* and *S. epidermidis* were the most prevalent Staphylococci associated with debris collected from the crew's quarters on the ISS (Venkateswaran et al. (2014). In addition, 13 *E. faecalis* and eight *B. cereus* strains were isolated from the crew and air-filters on the ISS (Schiwon et al., 2013). Taking the data of this study and others together (Van Houdt et al., 2012; Schiwon et al., 2013; Venkateswaran et al., 2014; Mayer et al., 2016) it can be concluded that the bacteria that survived on the different surfaces were predominantly human-associated.

Microbial diversity on the test materials increased over time. After 6 months only Staphylococci and Bacilli were found, after 12 months Staphylococci, Bacilli, *E. faecalis* and one *P. polymyxa* strain were isolated while after 19 months, Staphylococci, Bacilli, *E. aerosaccus, M. osloensis, M. yunnanensis*, and *P. psychrotolerans* were recovered. Novikova (2004) reported a similar diversity on surfaces on the MIR station including Staphylococci, Bacilli, *Micrococcus, Moraxella*, and *Pseudomonas*.

A decline of the number of Gram-positive human-pathogens recovered from V2A (*n* = 39) to V2A-Ag (*n* = 28) to V2A-AGXX® (*n* = 8) was observed. In total, only 12 bacteria were recovered from AGXX®-coated surfaces after 12 and 19 months exposure. AGXX® showed a pronounced antimicrobial effect, it reduced the microbial load by 79.5%. Silver also had a slight antimicrobial effect, it reduced the microbial load by 28.2%.

The antimicrobial test-materials are static surfaces, where dead cells, dust particles and cell debris can deposit. These deposits might interfere with the direct contact between the antimicrobial surface and the bacteria, which is required for effective antimicrobial activity of contact catalysts, such as Ag and AGXX®. Over time the deposits might have grown in size and thickness resulting in increasing interference with the antimicrobial activity. Possibly, this effect could explain that after 6 months no bacteria were recovered from AGXX®, whereas with prolonged exposure time a few bacteria escaped the antimicrobial action.

### Strong Biofilm Forming ISS Isolates

Biofilms provide microbes shelter from antimicrobials and the host immune system (Foulquié Moreno et al., 2006; Chen and Wen, 2011; Rafii, 2015; Qi et al., 2016; Hall and Mah, 2017). Bacterial biofilms have been associated with diseases such as cystic fibrosis, periodontitis, and nosocomial infections on catheters and prosthetic heart valves (Storti et al., 2005; Delle Bovi et al., 2011). Eradication of biofilms is difficult due to impaired penetration of antibiotics and the decreased host immune response. Thus, they can pose a health risk to immunosuppressed people, such as the crew on the ISS.

Most *Staphylococcus* and all *Enterococcus* isolates from this study formed strong biofilms. *B. cereus* isolates were more diverse in terms of biofilm formation: Seven isolates produced a weak, three a moderate and only one produced a strong biofilm. The fact that all bacterial isolates were able to form biofilms could be due to the long exposure to adverse space conditions.

### Prevalence of Antibiotic Resistances in Human Pathogenic Isolates

Astronauts have a suppressed immune response in-flight and as a consequence they are more susceptible to bacterial infections (Van Houdt et al., 2012; Taylor, 2015). The potential infection by pathogenic Staphylococci and Enterococci increases with duration of the mission (Schiwon et al., 2013). Therefore, treatability of bacterial infections on the ISS and on even longer space missions with limited amounts of antimicrobial drugs available is a health concern which has to be tackled.

In this study, all Gram-positive pathogenic isolates were resistant to at least one antibiotic. 68.0%, mostly Staphylococci, were multidrug resistant (resistant to more than three antibiotics). After 12 months exposure, also multi-resistant Enterococci occurred, one *E. faecalis* strain from V2A steel and two *E. faecalis* strains from V2A-AGXX®. *E. faecalis* V2A-12-03 had with nine resistances the largest number of resistances.

In total, the isolates were tested against 15 different antibiotics. Seven different antibiotic resistances were found after 6 months, 13 after 12 months and after 19 months, the number of resistances equalled the number after 6 months. This could be partly due to the fact, that the number of resistances in the Staphylococci declined after 19 months (most isolates had only one or two resistances), while *Bacillus* strains with more than three resistances came up.

All Staphylococci had similar antibiotic resistance profiles. The *B. cereus* isolates after 19 months exposure also showed similar resistance profiles. Most Bacilli and Staphylococci were resistant to ampicillin and erythromycin. Gentamicin resistance only occurred in *E. faecalis* isolates. Interestingly, all of them were also resistant to kanamycin. *E. faecalis* strains are known to be intrinsically resistant to low-level aminoglycosides (gentamicin, kanamycin) or have acquired high-level aminoglycoside resistance e.g., by uptake of *aac6-aph2a* or *aph(2)-ic* (Chow, 2000; Wendelbo et al., 2003; Dadfarma et al., 2013). As *E. faecalis* V2A-12-03 encodes *aac6-aph2a* and *E. faecalis* V2A-AGXX-12-02 and−03 encode *aph(2)-ic*, they are likely high level gentamicin resistant. *aac6-aph2a* was found on plasmids pSK41, pGO1, pLW1043, pSK1, pTEF1, on Tn*4001-*like transposons and on the chromosome (Schiwon, 2011). *aph(2)-ic* was found on conjugative plasmid pYN134 (Hollenbeck and Rice, 2012) in *E. gallinarum* but was shown to readily transfer to *E. faecalis* (Chow et al., 1997). Therefore, it is likely that gentamicin resistance spreads via these conjugative plasmids (Chow et al., 1997).

Most ISS-isolates were resistant to sulfamethoxazole, which interferes with bacterial synthesis of folic acid. It could be speculated that changes in the thickness of the cell wall due to exposure to space conditions might be involved in resistance to sulfamethoxazole by inhibiting the uptake of the antibiotic.

Most abundant resistance genes in the ISS-isolates were *ermC* and *tetK* coding for erythromycin and tetracycline resistance, respectively. Both genes are plasmid-borne and have been detected in Staphylococci of human origin (Schiwon et al., 2013). *ermC* was found on pSK41-like conjugative plasmid pUSA03 isolated from the community-acquired MRSA strain USA300 (Grohmann et al., 2003; Smillie et al., 2010; Schiwon et al., 2013). A pSK41-like plasmid could have spread *ermC* among the *S. aureus* strains V2A-6-13 and V2A-6-16, and between V2A-AG-6-03 and V2A-AG-6-04 isolated from the same material. Indeed, from *S. aureus* V2A-6-16 a plasmid >20 kbp was isolated. *ermB* is another plasmid-encoded erythromycin resistance gene. It is one of the 33 erythromycin resistance genes found in Staphylococci (Schiwon et al., 2013). However, *ermB* is not abundant in Staphylococci. No ISS-isolate from crew and air-filters harbored *ermB* (Zmantar et al., 2011; Schiwon et al., 2013). Also in this study, only *E. faecalis* V2A-AGXX-12-03 encoded *ermB*. De Leener et al. (2005) reported that *ermB* is present on Tn*1545*-like elements and that is likely associated with the occurrence of the tetracycline-resistance gene *tetM*. Interestingly, *E. faecalis* V2A-AGXX-12-03 harbored *tetM* along with *ermB*.

*tetK* is found on small mobilizable plasmids, which can be integrated into the *Staphylococcus* chromosome or into larger staphylococcal plasmids (Gillespie et al., 1987; Needham et al., 1994; Roberts, 2005). *tetO* and *tetK* can be found on pT181-like small mobilizable plasmids (Khan and Novick, 1983; Chopra and Roberts, 2001). *S. hominis* V2A-AGXX-12-01 (*tetK, tetO*) and *S. haemolyticus* V2A-AGXX-12-05 (*tetK*) likely carry pT181-like plasmids as small plasmid bands in the range of 2000–6000 bp were observed on the gel (data not shown). Both strains were isolated from the same material after the same time-period. Thus, the resistance genes might have spread via HGT among them. Along with *tetK*, pT181-like plasmids can carry *tetL* as well (Chopra and Roberts, 2001). Both genes were found in *S. hominis* V2A-AGXX-12-01.

Kanamycin resistance occurred both in Staphylococci and Enterococci. The kanamycin-resistance gene *aph3-III* was found in *S. hominis* V2A-12-04, *S. hominis* V2A-AG-12-05 and *E. faecalis* V2A-12-03, *E. faecalis* V2A-AGXX-12-02, and *E. faecalis* V2A-AGXX-12-03, all isolated from V2A and the two antimicrobial surfaces after 12 months. *aph3-III* is located on transposons of the Tn*916*-Tn*1545* type encoding a broad spectrum of resistances, toward tetracycline, macrolides, lincosamides, streptogramins, and kanamycin (Fons et al., 1997; Soge et al., 2008; Roberts and Mullany, 2011). The kanamycin-resistance gene *aadD* was detected in *S. hominis* V2A-AG-12-03, V2A-AG-12-05 and in *E. faecalis* V2A-AGXX-12-02 and V2A-AGXX-12-03. *aadD* is encoded on *S. aureus* plasmid pUB110 (4548 bp) (McKenzie et al., 1986; Allignet et al., 1998). As the two *S. hominis* and two *E. faecalis* strains were isolated from the same materials, V2A-Ag and V2A-AGXX®, respectively, transfer of the *aadD* gene might have taken place. *S. hominis* V2A-AG-12-05 showed plasmid-bands in the range of 2000-3000 bp and around 7000 bp likely indicating the presence of pUB110-like plasmids (data not shown). Occurrence of *aph3-III* and *aadD* genes in *Staphylococcus* and *Enterococcus* isolates from the ISS has already been reported (Schiwon et al., 2013).

### Antibiotic Resistance Transfer of the ISS-Isolates

Plasmids are the key players in HGT of antibiotic resistances (Kohler et al., 2018). Twenty multidrug-resistant, biofilm forming human-pathogenic staphylococcal isolates obtained from the three different materials after 6, 12, and 19 months were applied to plasmid DNA isolation. All isolates harbored plasmids <20 kbp and 17 of them also harbored plasmids >20 kbp. Commonly, *S. aureus* strains contain one or more plasmids ranging in size from <2000 bp to >60 kbp (Kwong et al., 2008).

Fourteen of the 17 *Staphylococcus* isolates with large plasmids were applied as donors to biparental matings to test the transferability of tetracycline and erythromycin resistance. In total, six out of seven tetracycline resistance transfer experiments (*S. hominis* V2A-6-05, *S. hominis* V2A-6-06, *S. haemolyticus* V2A-12-08, *S. hominis* V2A-AG-12-06, *S. hominis* V2A-AGXX-12-01, and *S. haemolyticus* V2A-AGXX-12-05) were successful whereas nine out of 18 erythromycin resistance transfer experiments (*S. hominis* V2A-6-03, *S. hominis* V2A-6-06, *S. hominis* V2A-6-11, *S. aure*us V2A-6-13, *S. aureus* V2A-6-14, *S. hominis* V2A-12-04, *S. haemolyticus* V2A-12-08, *S. hominis* V2A-AG-12-06, and *S. hominis* V2A-AGXX-12-06) were successful. Thus, these nine isolates likely harbor conjugative elements encoding erythromycin resistance. Indeed, in *E. faecalis* OG1X transconjugants of four of these matings large plasmids similar in size to those of the donors were found. pSK41 (46.4 kbp) and pUSA03 (37 kbp) are well known staphylococcal conjugative plasmids. Both carry *ermC* (Berg et al., 1998; Kennedy et al., 2010; Smillie et al., 2010) which was also detected in five of the successful donors.

Tetracycline resistance transfer frequencies from *S. hominis* V2A-6-05 (*tetK*), *S. hominis* V2A-6-06 (*tetK, tetO*), *S. haemolyticus* V2A-12-08 (*tetK*), *S. hominis* V2A-AG-12-06 (*tetK*), *S. hominis* V2A-AGXX-12-01 (*tetK, tetL*), *S. haemolyticus* V2A-AGXX-12-05 (*tetO*) to *S. aureus* 04-02891 ranged from 1.2 × 10^−7^ to 6.8 × 10^−4^ transconjugants/recipient. *tetK* is only rarely found on large staphylococcal plasmids. It is rather encoded on small mobilizable staphylococcal plasmids in the size range of 4.4 to 4.7 kbp, such as pT181 (Chopra and Roberts, 2001). Thus, in the successful matings with donors harboring *tetO* or *tetK* mobilizable pT181-like plasmids might have played a role in the transmission of the resistance to *S. aureus* 04-02981. As pT181 is non self-transmissible another conjugative element has participated in the transfer of the tetracycline resistance. All donors that were successful in the tetracycline resistance matings contained in addition to plasmid-bands <20 kbp at least one plasmid-band >20 kbp, which could represent the conjugative plasmid. Thus, it is likely that the successful donors harbor a pT181-like plasmid which was transferred by the help of a conjugative plasmid. Indeed, in *S. aureus* 04-02981 transconjugants from all of those matings large plasmids similar in size to those of the donors were detected. In addition, small plasmids in the size range of pT181-like plasmids were found in transconjugants of three of these matings.

Transfer frequency of gentamicin resistance (8.3 × 10^−4^ transconjugants/recipient) from *E. faecalis* V2A-12-03 (*aac6-aph2a*) to *S. aureus* 04-02891 was higher than from *E. faecalis* V2A-AGXX-12-03 (*aph(2)-ic*) to the same recipient (9.2 × 10^−7^ transconjugants/recipient). *aac6-aph2a* can be found on conjugative plasmids, such as pSK41, pGO1, pLW1043, pSK1, pTEF1, Tn*4001*-like transposons but also on the chromosome (Schiwon, 2011). The uptake of *aac6-aph2a* by *S. aureus* 04-02891 indicates that *E. faecalis* V2A-12-03 likely harbors one of these conjugative elements. Indeed, this observation was corroborated by isolation of a plasmid >20 kbp from *E. faecalis* V2A-12-03. *aph(2)-ic* was found on the 34-kbp conjugative plasmid pYN134 (Chow et al., 1997; Hollenbeck and Rice, 2012). The uptake of *aph(2)-ic* by *S. aureus* 04-02891 suggests that *E. faecalis* V2A-AGXX-12-03 likely harbors a pYN134-like plasmid. This argument was corroborated by the observation of a plasmid band >20 kbp for *E. faecalis* V2A-AGXX-12-03.

The data of this study confirm erythromycin and tetracycline resistance transfer in ISS-isolates from air-filters and the crew as reported by Schiwon et al. (2013). Further transfer studies between ISS-isolates could deepen our knowledge in the transmissibility of antibiotic resistances. However, no methicillin resistant Staphylococci and no vancomycin resistant enterococci were found. Thus, the generation of serious multi-resistant pathogens by horizontal transfer is unlikely.

### Further Applications of the Antimicrobial Surface

AGXX® proved to be a long-term efficient antimicrobial, even under the harsh conditions on the ISS. The antimicrobial coating has been also successfully applied against other Gram-positive and Gram-negative pathogens. It also strongly reduced the bacterial load of *Legionella* and the highly pathogenic Shiga toxin-producing *E. coli* O104:H4 strain (Guridi et al., 2015). It is available in diverse application forms, such as powders, thin sheets, as coating on diverse plastic materials and on cellulose fleece and will be recently tested in the 4 months SIRIUS isolation study for future lunar flights.

## Data Availability

All datasets generated for this study are included in the manuscript and/or the supplementary files.

## Author Contributions

EG designed the project and supervised all the experiments. L-YS, KR, JF, WS, PO, and AV performed the experiments. L-YS, KR, and EG wrote the manuscript and designed the figures and tables. NN provided us access to the BIORISK experiment on the ISS and contributed with insightful discussions on the experimental design. All authors read and revised the manuscript.

### Conflict of Interest Statement

The authors declare that the research was conducted in the absence of any commercial or financial relationships that could be construed as a potential conflict of interest.
