# Supplementary material for: Biofilm Forming Antibiotic Resistant Gram-Positive Pathogens Isolated From Surfaces on the International Space Station
Source: Front Microbiol. 2019 Mar 19;10:543. doi: 10.3389/fmicb.2019.00543 (PMC6433718; doi:10.3389/fmicb.2019.00543)
Supplement: Supplementary file 1 [file Table_1.docx]

Supplementary Table 1: PCR-programs for antibiotic resistance genes (AB-R genes)

| No. | AB-R gene | *aac6-aph2a, aph(2)-Ib/Ic/Id, aph3-III, ermB, ermD, ermG* | | | | | |
| --- | --- | --- | --- | --- | --- | --- | --- |
| 1 |  | T [°C] | t [m:s] | T [°C] | t [m:s] | T [°C] | t [m:s] |
|  | Denaturation | 95 | 02:00 | 95 | 00:30 |  |  |
|  | Annealing |  |  | 56 | 00:30 |  |  |
|  | Elongation |  |  | 72 | 00:30 | 72 | 05:00 |
|  | Cycles | 1 | 30 | 1 |  |  |  |
| No. | AB-R gene | *aadD* | | | | | |
| 2 |  | T [°C] | t [m:s] | T [°C] | t [m:s] | T [°C] | t [m:s] |
|  | Denaturation | 95 | 02:00 | 95 | 00:30 |  |  |
|  | Annealing |  |  | 62 | 00:30 |  |  |
|  | Elongation |  |  | 72 | 00:45 | 72 | 05:00 |
|  | Cycles | 1 | 30 | 1 |  |  |  |
| No. | AB-R gene | *sul1, sul2* | | | | | |
| 3 |  | T [°C] | t [m:s] | T [°C] | t [m:s] | T [°C] | t [m:s] |
|  | Denaturation | 95 | 02:00 | 95 | 00:30 |  |  |
|  | Annealing |  |  | 60 | 00:30 |  |  |
|  | Elongation |  |  | 72 | 00:45 | 72 | 05:00 |
|  | Cycles | 1 | 30 | 1 |  |  |  |
| No. | AB-R gene | *ampC, tetK, tetM, tetO, tetS, tetL* | | | | | |
| 4 |  | T [°C] | t [m:s] | T [°C] | t [m:s] | T [°C] | t [m:s] |
|  | Denaturation | 95 | 02:00 | 95 | 00:30 |  |  |
|  | Annealing |  |  | 55 | 00:30 |  |  |
|  | Elongation |  |  | 72 | 00:30 | 72 | 05:00 |
|  | Cycles | 1 | 30 | 1 |  |  |  |
| No. | AB^R^-gene | *mecA* | | | | | |
| 5 |  | T [°C] | t [m:s] | T [°C] | t [m:s] | T [°C] | t [m:s] |
|  | Denaturation | 95 | 02:00 | 95 | 00:30 |  |  |
|  | Annealing |  |  | 60 | 00:30 |  |  |
|  | Elongation |  |  | 72 | 00:30 | 72 | 05:00 |
|  | Cycles | 1 | 30 | 1 |  |  |  |
| No. | AB-R gene | *blaZ, blaSHV-5* | | | | | |
| 6 |  | T [°C] | t [m:s] | T [°C] | t [m:s] | T [°C] | t [m:s] |
|  | Denaturation | 95 | 02:00 | 95 | 00:30 |  |  |
|  | Annealing |  |  | 60 | 00:45 |  |  |
|  | Elongation |  |  | 72 | 00:30 | 72 | 05:00 |
|  | Cycles | 1 | 30 | 1 |  |  |  |
| No. | AB-R gene | *ermA, ermC* | | | | | |
| 7 |  | T [°C] | t [m:s] | T [°C] | t [m:s] | T [°C] | t [m:s] |
|  | Denaturation | 95 | 02:00 | 95 | 00:30 |  |  |
|  | Annealing |  |  | 56 | 00:30 |  |  |
|  | Elongation |  |  | 72 | 00:30 | 72 | 05:00 |
|  | Cycles | 1 | 30 | 1 |  |  |  |
